# Minocycline Ameliorates Depressive-Like Behavior and Demyelination Induced by Transient Global Cerebral Ischemia by Inhibiting Microglial Activation

**DOI:** 10.3389/fphar.2019.01247

**Published:** 2019-10-22

**Authors:** Bingying Du, Hailong Li, Huiwen Zheng, Cunxiu Fan, Meng Liang, Yongjie Lian, Zelan Wei, Yanbo Zhang, Xiaoying Bi

**Affiliations:** ^1^Department of Neurology, Shanghai Changhai Hospital, the Second Military Medical University, Shanghai, China; ^2^Department of Neurology, General Hospital of Central Theater Command of Chinese People's Liberation Army, Wuhan, China; ^3^Department of Rehabilitation Medicine, Zhejiang Hospital, Hangzhou, China; ^4^Department of Psychiatry, College of Medicine, University of Saskatchewan, Saskatoon, SK, Canada

**Keywords:** cerebral ischemia, oligodendrocyte, myelin, microglia, minocycline, vascular depression, inflammation

## Abstract

Global cerebral ischemia (GCI) commonly occurs in the elderly. Subcortical white matter lesions and oligodendrocyte (OLG) loss caused by cerebral ischemia have been implicated in the development of post-ischemic depression and cognitive impairment. OLGs are necessary for axonal myelination; the disrupted differentiation of OLG progenitor cells (OPCs) is associated with impaired remyelination. Evidence has indicated that increased levels of inflammatory cytokines released from activated microglia induce depression-like behaviors by affecting neurotransmitter pathways, but the mechanisms remain elusive. We explored the potential mechanisms that link microglia activation with GCI-induced depression and cognitive dysfunction by studying effects of minocycline on white matter damage, cytokine levels, and the monoaminergic neurotransmitters. An acute GCI animal model was generated through bilateral common carotid artery occlusion to induce ischemic inflammation and subcortical white matter damage. Minocycline, an inhibitor of microglia activation, was intraperitoneally administrated immediately after surgery and continued daily for additional six days. Minocycline shortened the immobile duration in tail suspension test and forced swimming test, while no improvement was found in Morris water maze test. The plasma levels of IL-1β, IL-6, TNF-α, HMGB1, and netrin-1 were significantly reduced with the treatment of minocycline. Minocycline treatment substantially reversed demyelination in corpus callosum and hippocampus, alleviated hippocampal microglia activation, and promoted OPCs maturation, while no effect was found on hippocampal neurodegeneration. Besides, the content of dopamine (DA) in the hippocampus was upregulated by minocycline treatment after GCI. Collectively, our data demonstrated that minocycline exerts an anti-depressant effect by inhibiting microglia activation, promoting OPCs maturation and remyelination. Increased DA in hippocampus may also play a role in ameliorating depressive behavior with minocycline treatment.

## Introduction

Cerebral ischemia is a leading cause of neurological disability and mortality in the elderly (Feigin et al., 2014; Mori et al., 2017). It occurs when the blood supply of the brain is lost due to blockage of a brain artery (ischemic stroke) or cerebral hypoperfusion from cardiac arrest or cardiac surgery (Chu et al., 2012). People who survived cerebral ischemia may show persistent neurological, emotional, and cognitive symptoms (Torgersen et al., 2010; Alexander et al., 2011). A meta-analysis has shown that vascular depression (VD) and vascular cognitive impairment (VCI) are the most prevalent neuropsychiatric conditions after cerebral ischemia (Hackett and Pickles, 2014). New medications and interventions are needed as current pharmacological therapies for dementia and depression and are less effective in treating patients with VD or VCI (Iadecola and Anrather, 2011; Dirnagl and Endres, 2014).

Studies have indicated that white matter injury and oligodendrocyte (OLG) loss are closely associated with depressive symptoms and cognitive dysfunction in the animal models of stroke (Bi et al., 2012; Ma et al., 2015). Subcortical white matter pathology is a characteristic feature in patients with VD or VCI (Krishnan et al., 1997; Pimontel et al., 2013; Hase et al., 2018). OLGs provide the axonal myelination and maintain the integrity of neural connectivity in the central nervous system (CNS) (Bradl and Lassmann, 2010). OLGs are vulnerable to ischemia and inflammation which eventually will induce cell death and demyelination (Hamilton et al., 2016). Microglia activation in acute and chronic ischemic status is key to the exacerbation of depressive symptoms in VD and deterioration of memory function in VCI (Disabato and Sheline, 2012; Paradise et al., 2012; Qin et al., 2017). Activated microglia can be categorized into M1 and M2 phenotypes (Qin et al., 2017; Han et al., 2018). M1 microglia are considered to be pro-inflammatory and the primary source of IL-1β, IL-6, and TNF-α (Gregersen et al., 2000; Ceulemans et al., 2010), which contribute to demyelination and neurodegeneration caused by stroke (Becker et al., 2011). Increased levels of these cytokines could also induce depression-like behavior by affecting neurotransmitter pathways (Miller, 2009; O’Connor et al., 2009). M2 microglia are considered to be anti-inflammatory and ameliorate inflammation-induced demyelination and neurodegeneration (Yu et al., 2015).

Minocycline is a second-generation tetracycline that can selectively inhibit microglia activation and M1 polarization both *in vivo* and *in vitro* (Kobayashi et al., 2013). Previous studies have reported that minocycline reduced white matter damage and improved cognitive function after focal or global cerebral ischemia (Yrjanheikki et al., 1999). Studies have also shown promising antidepressant effects of minocycline in clinical trials and animal models of depression (Burke et al., 2014; Rosenblat and McIntyre, 2018). However, the antidepressant effect of minocycline in treating depressive symptoms caused by GCI is unknown. The current study sought to examine whether minocycline could alleviate white matter damage and ameliorate depression or cognitive impairment behaviors in a GCI animal model through inhibiting microglia activation. We tested the effects of minocycline on monoaminergic neurotransmitters levels as well.

## Materials and Methods

### Surgery

In this study, we generated an acute GCI animal model by using a bilateral common carotid artery occlusion (BCCAO). We and others have shown that BCCAO induces depression and cognitive impairment-like behaviors, subcortical white matter damage, and neuroinflammation in the mouse model (Bi et al., 2012; Miyamoto et al., 2013; Ma et al., 2015; Soares et al., 2016; Mori et al., 2017). In this model, activated microglia and reactive astrocytes are present within the lesion sites (Kim et al., 2011; Bi et al., 2012). Furthermore, neuroinflammation induced by activated microglia after ischemia–hypoxia is an important factor involved in white matter damage and OLG death (Su et al., 2011; Jalal et al., 2012; Mori et al., 2017), indicating that microglia play an important role in demyelination following transient GCI. Therefore, the depressive behavior and demyelination following transient cerebral ischemia, such as clinical transient ischemic attack (TIA), can be studied using a transient, intermittent BCCAO mouse model. In this study, we applied a previously described BCCAO procedure with some modifications (Bi et al., 2012). Briefly, mice were anesthetized during the entire procedure with an isoflurane anesthesia system. Both common carotid arteries were exposed and occluded with cotton threads for 5 min, and then, threads were removed; 10 min later, arteries were occluded with cotton threads for another 5 min. Mice in the sham group received the same procedures except for the occlusion. All mice were then placed under a small animal heating lamp to prevent postsurgical hypothermia.

### Experimental Design

**Figure 1:**
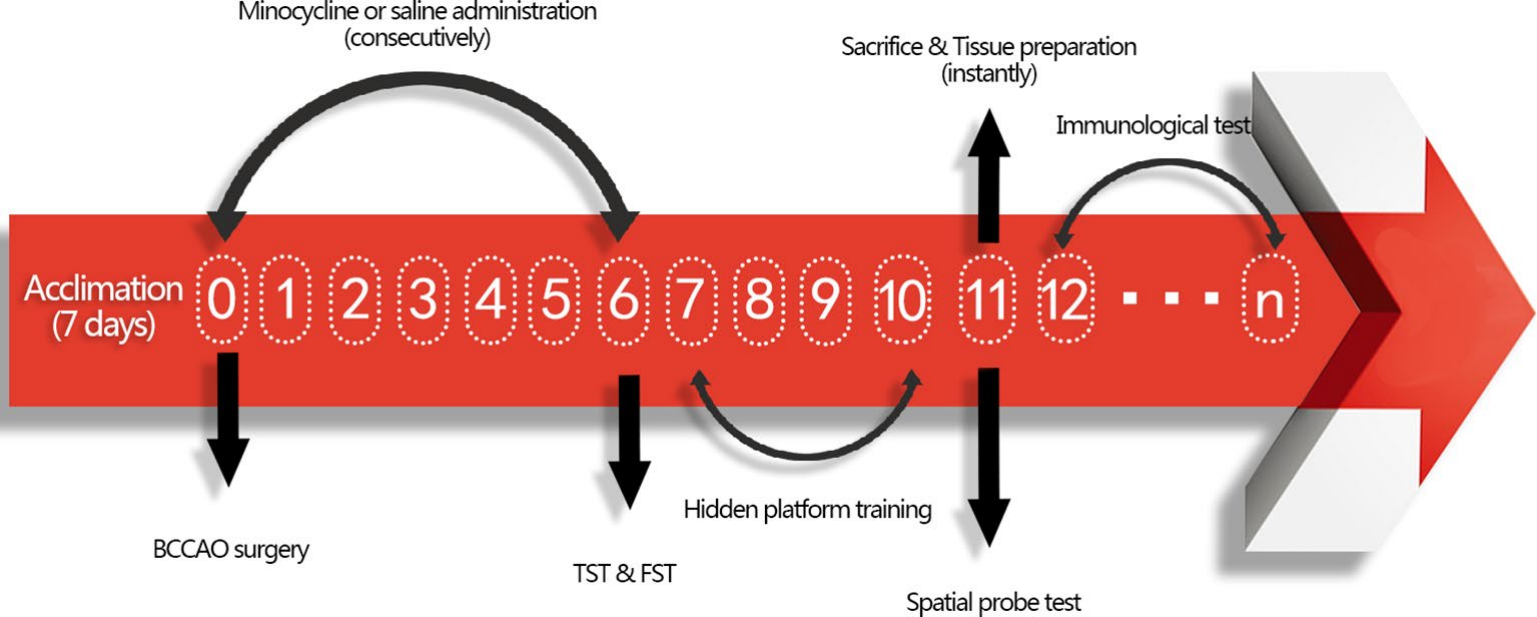
Flow chart for the experimental design. Mice were firstly acclimated for 7 days, and then BCCAO or sham surgery was conducted on POD 0. After administration of minocycline or saline for consecutive 7 days, tail suspension test (TST) and forced swimming test (FST) were performed to exam antidepressant function of minocycline on POD 6. From POD 7-10, mice were trained to find hidden platform daily and tested by spatial probe test on POD 11. Tissue was collected instantly after sacrifice, including blood extraction and brain tissues collection. Afterwards, immunological tests were conducted from POD12.

All procedures were approved by the Animal Care Committee of the Second Military Medical University and in accordance with the Animal Research Guidelines for the Care and Use of Laboratory Animals. The mice were housed under standard laboratory conditions (temperature 22 ± 1°C; humidity 52 ± 2%; 12 h day/night rhythm) with food and water available. Thirty-two male ICR mice (28–32 g, purchased from the animal center at the Second Military Medical University, China) were randomly clustered into three groups after 1 week of acclimation: sham group with normal saline (NS) treatment (sham + NS, n = 10), GCI group with NS treatment (GCI + NS, n = 11), and GCI with minocycline (MIN) treatment (GCI + MIN, n = 11). BCCAO surgery was conducted at 9:00 am, and this date was defined as post-operation day (POD) 0. After surgery, there was one animal loss in GCI+NS and GCI+MIN group. MIN (30 mg/kg in saline) or saline was administrated intraperitoneally immediately after BCCAO surgery and in the following 6 days (POD0-POD6) with the same dosage on a daily base. Behavioral tests to assess the antidepressant effect of MIN were carried out on POD6. The behavioral tests to measure cognitive function were conducted from POD7 to POD11. After behavioral tests, mice were sacrificed for sample collection. The flow chart for the experimental design was showed in **Figure 1**.

### Behavioral Tests

All behavioral tests were performed during the dark phase (19:00–20:00) (Lian et al., 2017).

#### Tail Suspension Test

Tail suspension test (TST) is one of the widely used tests to assess the antidepressant function of drugs. In this test, mice were subjected to an inescapable aversive situation. The alternation between mobile (struggling behavior) and immobile (waiting behavior) periods reflects “behavioral despair” (Steru et al., 1985). According to our previous study (Lian et al., 2017), mice were suspended with tails taped to the edge of a shelf that was 30 cm above a tabletop for 6 min. The duration of immobility, which reflected the depressive state, was recorded during the last 4 min by a computer-based system (MED Associates, America).

#### Forced Swimming Test

Forced swimming test (FST) was developed by Porsolt et al. (1977) as an assay of depressive-like behavior. Each mouse was placed into a Plexiglas cylinder (30 cm height by 20 cm diameter) filled with water in 25 ± 1°C (20 cm high from the bottom of the cylinder) for 6 min (Haj-Mirzaian et al., 2016). The immobility was defined as the mouse only showing minimal movement to keep the head above the water. The immobile time during the last 4 min was measured using a video camera and analyzed by two researchers who were blind to the treatment. After sessions, mice were carefully dried and returned to their home cages. Water was changed between subjects.

#### Morris Water Maze

The hippocampal-dependant spatial learning and memory were tested using the hidden platform and spatial probe test in the Morris water maze (MWM) from POD7 to POD11 as previously described (Vorhees and Williams, 2006). In short, the circular tank was divided into four quadrants, and a 6-cm-diameter hidden platform was submerged 2 cm below the water surface in the northwest as target quadrant. During each trial, mice were released from one of the three quadrants to search for the platform in the target quadrant with total three trials per day from POD7 to POD10. The escape latency, the duration of time each mouse sought the platform, was recorded using a video camera (Xinruan Technology Co., Ltd, Shanghai, China). If mice failed to find the platform within 60 seconds, they were gently guided to the platform and allowed to rest on it for 10 s. For spatial probe test on POD11, the platform was removed from the pool, and mice were placed into the pool for 60 s. The swim speed, the time spent and the distance travelled in the target quadrant, and the number of crossings of the target quadrant were recorded.

### Tissue Preparation

Blood samples and brain tissues were collected after the MWM test (POD11). Mice were anesthetized with sodium pentobarbital (75 mg/kg). The blood sample was extracted from retro-orbital sinus using a heparinized capillary tube (I.D 1 mm). After blood sample extraction, mice were perfused intracardially with phosphate buffered saline (PBS) and then decerebrated. The left hemisphere was immediately post-fixed with 4% paraformaldehyde for later use in immunohistochemistry study. The right hippocampus was dissected, freshly frozen at -80°C for later used in HPLC and western blot analysis.

### Immunohistochemistry

Brain tissues were cut into 30 μm serial sections, rinsed with PBST (0.1 M PBS, pH 7.4, plus 3% Triton X-100) three times, and incubated in PBS with 1% H_2_O_2_ for 30 min to block the endogenous peroxidase activity. Sections were rinsed with PBST and incubated in PBS with 2% bovine serum albumin (BSA) for 60 min at room temperature. Sections were then incubated with antibodies in the same solution including either anti-ionized calcium-binding adapter-1(Iba-1; Wako Cat# 019-19741), or anti-myelin basic protein (MBP; Santa Cruz Biotechnology Cat# sc-13914), or anti-nerve/glial antigen 2 (NG2; Millipore Cat# MAB5384), or anti- neuron-specific nuclear-binding protein (NeuN; Giosobio Cat# GB11138, 1:200) at 4°C overnight. Sections were washed with PBST three times, incubated with biotinylated goat anti-rabbit secondary antibody (Abcam Cat# ab6721) at 37°C for 30 min, and then visualized by using 3,3-diaminobenzidine (DAB) kit (Zhongshan Gold Bridge Biology Company, Beijing, China). Sections were counterstained with hematoxylin. Luxol fast blue (LFB) (Sigma, St. Louis, MO, USA) staining, which labels myelin sheath structures, was also used to confirm demyelination in corpus callosum (CC) as previously reported (Pappas, 1981). Briefly, dehydrated sections were immersed in 0.1% LFB staining solution (Sigma, USA) with acidified 95% ethanol overnight at 60°C. Sections were rinsed in 95% ethanol followed by distilled water. The differentiation of sections was obtained by immersing the slides in 0.05% Li_2_CO_3_ and 70% ethanol several times. The proper differentiation was confirmed under a microscope when sharp contrast was achieved between the gray matter and the white matter. Images were obtained using a digital microscope (Leica Microsystems, Berlin, Germany) and analyzed by a computerized image system (Quantimet 500 Image Processing and Analysis System, Qwin V0200B Software; Leica, Berlin, Germany).

LFB and MBP immunostaining were measured in three fields from the same levels and presented as integrated optical density (IOD) by average density multiplied by LFB- or MBP-positive area in the total scanned area. The positive cells were counted and presented as the number of per mm^2^. Images were acquired with a Zeiss Microscope (Zeiss Instruments Inc., Germany) and analyzed with the Image-Pro Plus software (version 6.0).

### Enzyme-Linked Immunosorbent Assay (ELISA)

The cytokine levels of IL-1β, IL-6, TNF-α, and HMGB1 and netrin-1 in plasma were measured (n = 10 in each group) using ELISA Kits (Westang Biotechnology Co., Ltd., Shanghai, China) according to the manufacturer’s protocol. Blood samples were allowed to clot at room temperature for half an hour and then centrifuged at 5,000 rpm for 10 min at 4°C. The supernatants were collected and stored at −80°C for subsequent ELISA analysis.

### Measurements of Dopamine (DA), Serotonin (5-HT), and Norepinephrine (NE) in the Hippocampus

Chromatographic analysis was performed on an Agilent Series 1100 Ultra-High Performance Liquid Chromatography (UHPLC) System (Agilent Technologies, Santa Clara, CA). Samples were chromatographically separated through an Agilent ZORBAXSB-C18 column (4.6 mm × 150 mm, 5 μm) with 0.5% aqueous formic acid (A)-menthol (B) in the mobile phase. The flow rate was maintained at 1.0 ml/min, and the injection volume was set at 20 μl. The standards of DA, 5-HT, and NE were shed in a buffer containing 20 mM Tris HCl (pH 7.5), dissolved in 2% HClO_4_ and then diluted to a series of concentrations (2.0,1.0, 0.4,0.2,0.1, and 0.04 μg/ml). Each sample (20 μl) was measured in a liquid scintillation counter.

### Western Blot

The western blot analysis was performed in order to semi-quantitative the protein levels of CD11b in the hippocampus. For immunoblotting, an equal amount (30 μg) of total protein from each sample was loaded onto a 12% SDS-polyacrylamide gel electrophoresis, and then, the separated proteins were transferred onto a polyvinylidene difluoride (PVDF) membrane (Millipore, CA). The membranes were blocked with 5% fat-free milk in TBS-T (TBS contained 0.1‰ Tween-20) for 60 min at RT and subsequently incubated overnight with primary antibodies including anti-CD11b (Abcam Cat# ab133357) and anti-β-actin (Abcam Cat# ab8227) at 4°C. After washed with TBS-T three times, membranes were incubated with HRP-conjugated secondary antibodies: anti-rabbit Ig-G (Santa Cruz Biotechnology Cat# sc-2357) for 60 min at RT. After three washes with TBS-T, bands on membranes were visualized in ECL system. The IODs were calculated using ImageJ Software (NIH) and standardized to β-actin levels in the same membrane.

### Statistical Analysis

Data were analyzed using one-way analysis of variance (ANOVA) followed by *post hoc* analysis using Fisher’s Least Squares Difference (LSD) tests. The level of significance was set at *P* < 0.05. All data were presented as the mean ± standard error of the mean (SEM) and analyzed using the GraphPad Prism 6 (GraphPad Software, Inc., La Jolla, CA).

## Results

### Minocycline Attenuated GCI-Induced Depression-Like Behavior

**Figure 2:**
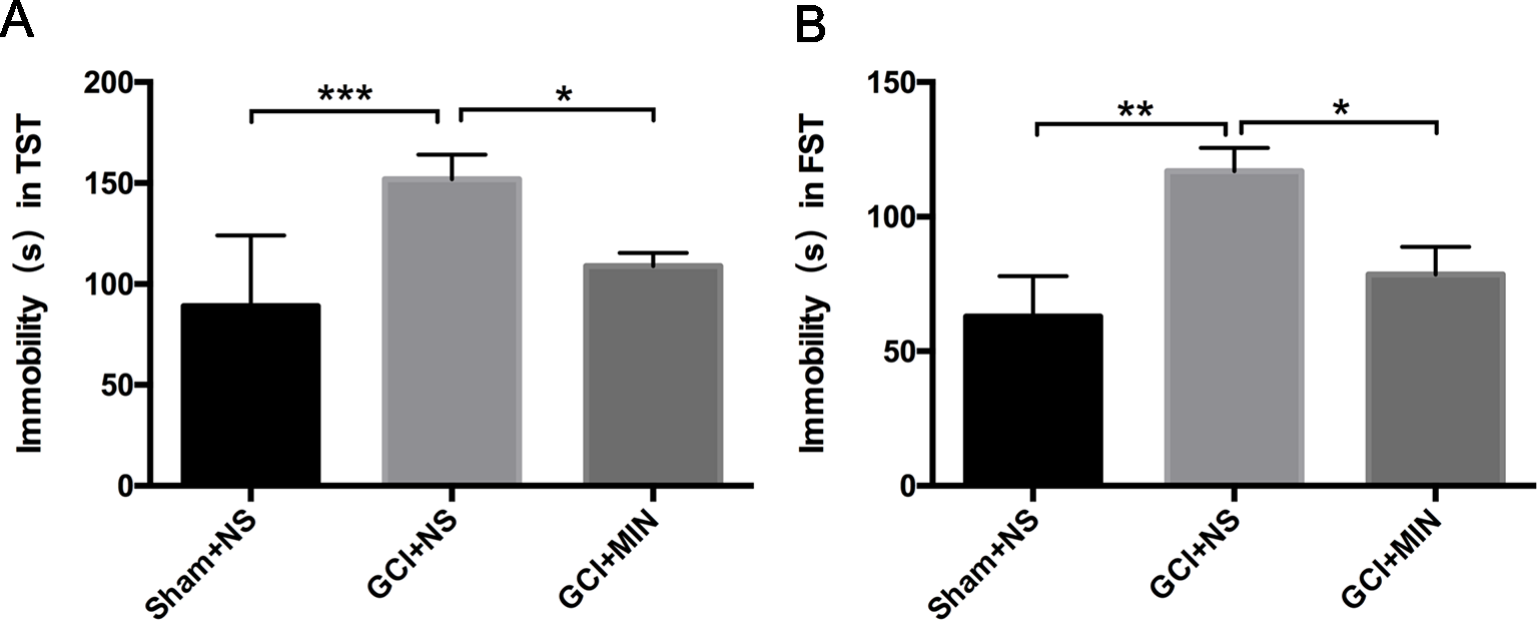
Minocycline improved the depressive behavior induced by GCI in mice. As revealed by TST **(A)** and FST **(B)** on POD6, GCI mice demonstrated depression-like behavior compared to sham mice with the longest immobility time among three groups. Minocycline-treatment shortens the immobility time of GCI mice in both of TST and FST. (*p < 0.05, **p < 0.01, ***p < 0.001, compared with GCI+NS, n = 10).

In TST and FST studies, one-way ANOVA indicated a significant difference among three groups, with mice in the GCI+NS group showing the longest immobile time among the three groups (**Figures 2A, B**, TST: *F* = 9.059, *P* = 0.001; FST: *F* = 5.969, *P* = 0.007). The significantly increased immobile time in GCI+NS mice *vs*. sham+NS mice was revealed by LSD *post hoc* test, indicating that GCI induced depression-like behavior (TST: *p* < 0.001, FST: *p* < 0.05). Administration of MIN decreased the immobile time in GCI+MIN mice in both tests (TST: *p* = 0.014, FST: *p* = 0.023), demonstrating that MIN ameliorated the depression-like behaviors in mice caused by GCI.

### Minocycline Did Not Reverse GCI-Induced Cognitive Impairment

**Figure 3:**
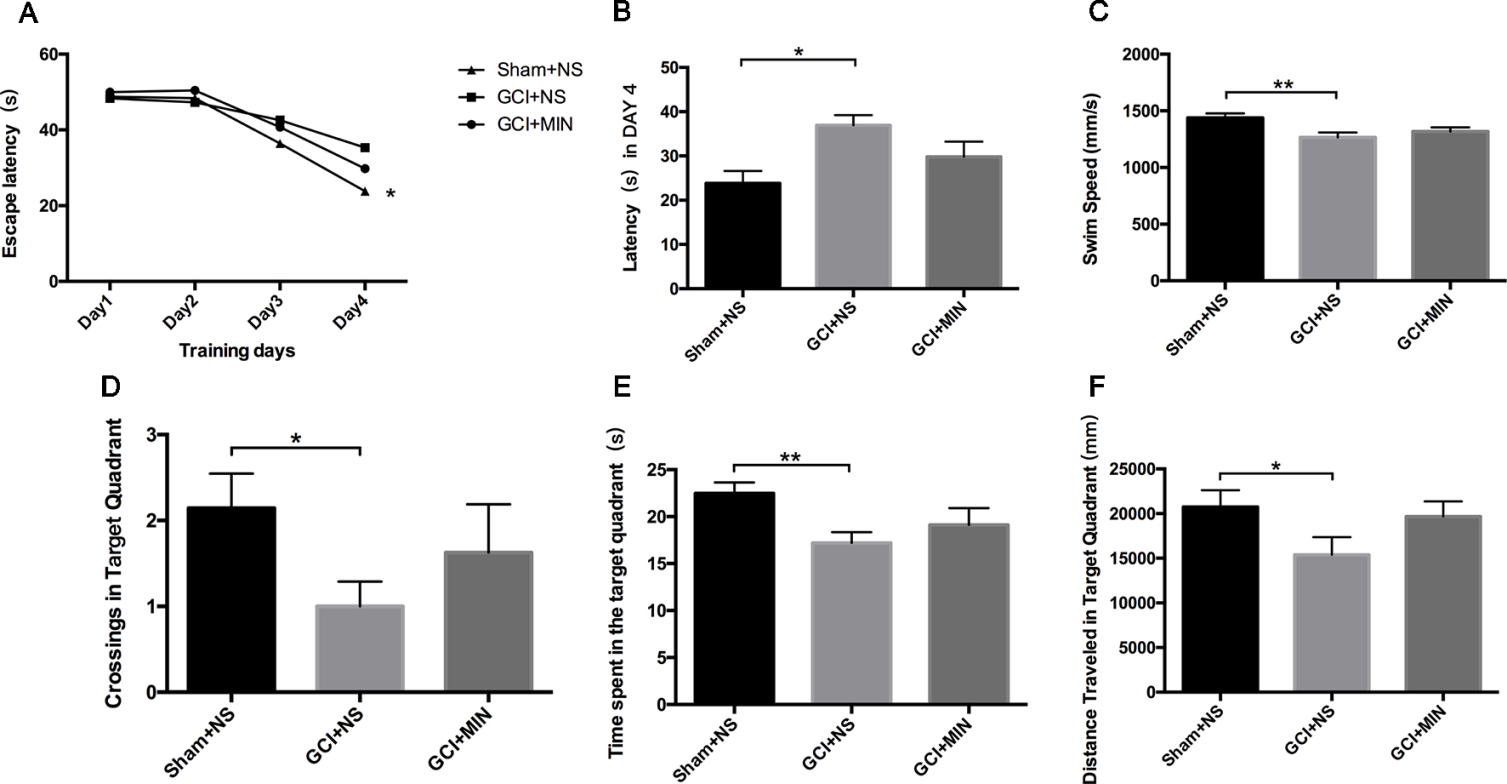
Minocycline did not improve the cognitive impairment induced by GCI in mice. Learning curve of MWM represented poor spatial learning and memory of GCI animals **(A)**. Minocycline treatment did not decrease latency on POD10 **(B)** or increase swim speed **(C)**, crossings **(D)**, time spent **(E)**, as well as distance travelled **(F)** in the target quadrant on POD11. (*p < 0.05, **p < 0.01, compared with GCI + NS, n = 10).

The effect of MIN on hippocampal-dependent spatial learning and memory was tested by MWM. **Figure 3A** showed the escape latencies on training sessions of 4 days (POD7-10). On the 4th day, a significant *model-by-treatment* interaction (*F* = 3.743, *P* = 0.037) was seen with the GCI+NS group showing noticeable longer latency compared to the sham+NS group (**Figure 3B**, *p* = 0.011), indicating reduced learning and memory ability occurred in GCI. Mice in GCI+MIN group showed a tendency to shortened latency compared to GCI+NS group; however, the difference did not reach the statistical significance (*p* = 0.188). Likewise, on the spatial probe test, the swim speed (*F* = 6.559, *P* = 0.005), the number of crossing target quadrant (*F* = 2.323, *P* = 0.122), the distance travelled (*F* = 2.783, *P* = 0.080) and the time spent in the target quadrant (*F* = 4.100, *P* = 0.028) were calculated and analyzed by ANOVA. All the four measures were significantly different between GCI+NS and sham+NS groups (**Figures 3C–F**, *p* = 0.001, 0.042, 0.038, and 0.008, respectively). However, none of these four measures were significantly different between GCI+NS and GCI+MIN groups (*p* = 0.331, 0.293, 0.081, and 0.249, respectively), indicating the limited efficacy of MIN to reverse the cognitive impairment in mice with GCI.

#### Minocycline Reversed GCI-Induced Demyelination in CC and Hippocampus

**Figure 4:**
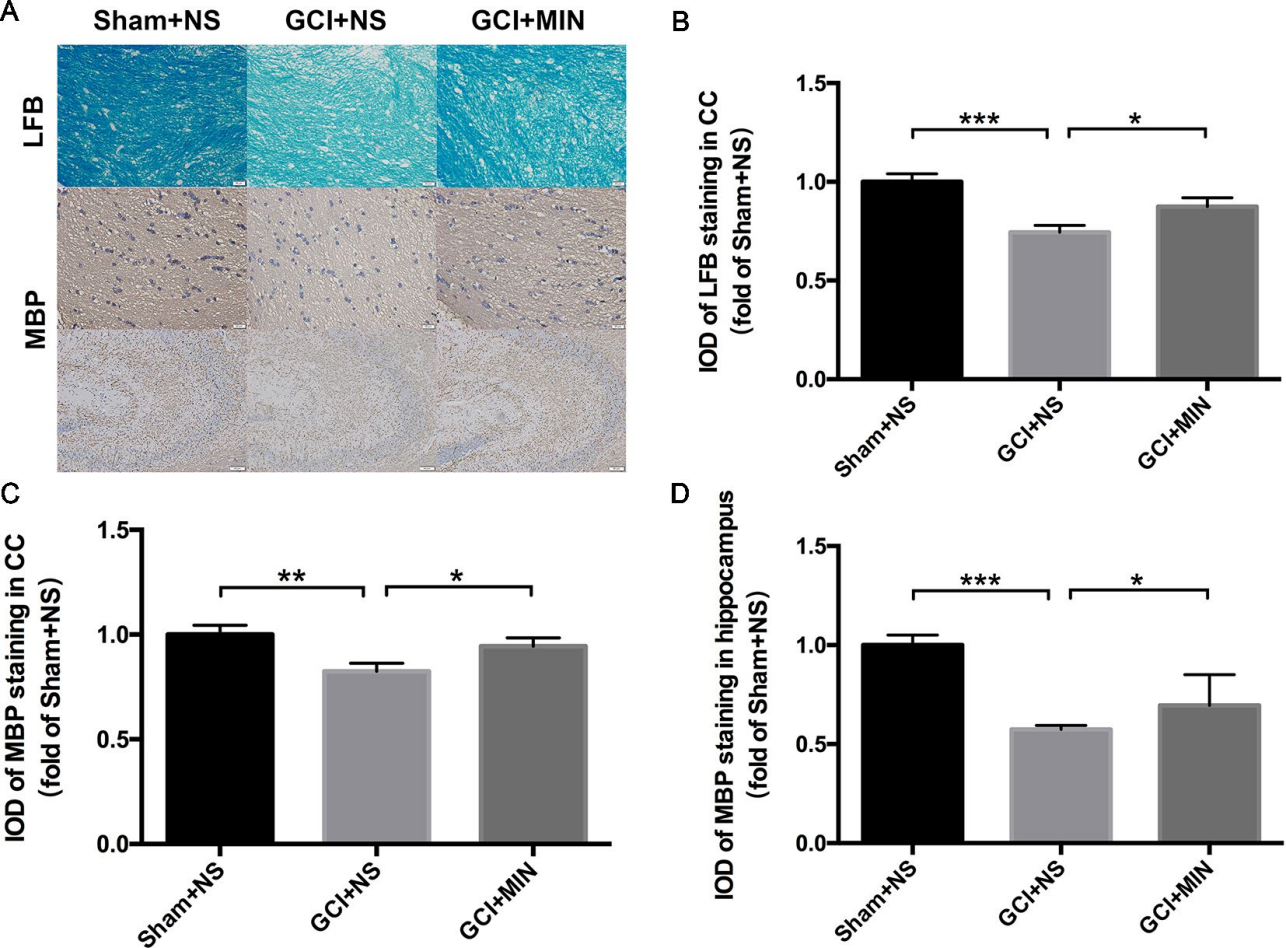
Minocycline reversed demyelination of GCI-treatment mice in CC and hippocampus. As revealed by immunohistochemistry of myelinated OLGs in CC and hippocampus, GCI surgery induced significant demyelination, while minocycline administration reversed the effect. **(A)** shows the representative photographs of LFB staining in CC and MBP staining in CC and hippocampus. **(B**–**D)** show the above three statistical results of IOD scores, respectively. (*p < 0.05, **p < 0.01, ***p < 0.001 compared with GCI + NS, n = 10).

LFB staining and MBP immunohistochemistry staining in the CC and hippocampus were applied for detecting the demyelination as shown in **Figure 4A**. One-way ANOVA indicated significant differences in the IOD of LFB and MBP staining among the three groups (LFB in CC: *F* = 10.523, *P* < 0.001; MBP in CC: *F* = 4.831, *P* = 0.016; MBP in hippocampus: *F* = 32.128, *P* < 0.001). *Post hoc* comparisons revealed that IOD scores of LFB and MBP staining in GCI+NS group were significantly lower than those in sham+NS group (LFB in CC: *p*<0.001; MBP in CC: *p*<0.001; MBP in hippocampus: *p* < 0.001), indicating that demyelination in CC and hippocampus occurred in GCI mice (**Figures 4B–D**). Importantly, MIN attenuated those IOD decreases in CC (LFB: *p* = 0.033; MBP: *p* = 0.047) and hippocampus (MBP: *p* < 0.012), indicating that MIN reversed GCI-induced demyelination in those areas.

#### Minocycline Inhibited the Microglia Activation and Decreased Levels of IL-1**β**, IL-6, TNF-**α**, HMGB1, and Netrin-1 in Plasma

**Figure 5:**
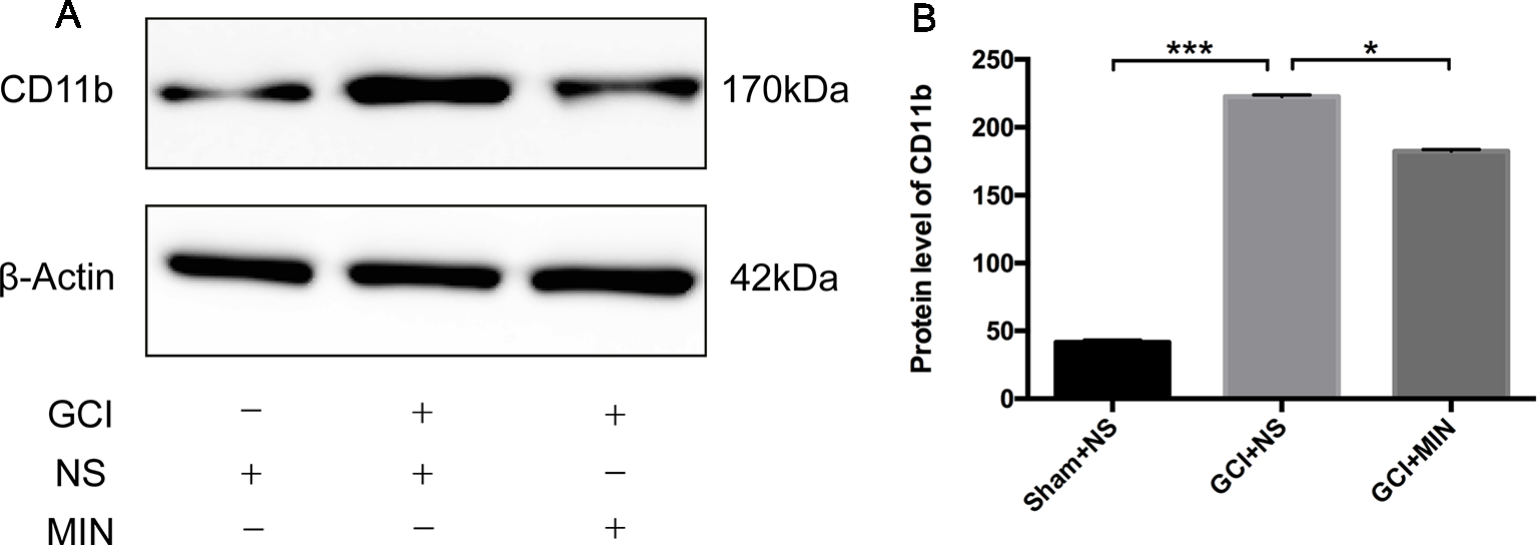
Western blot analysis of CD11b protein in the hippocampus. **(A)** Western blot of CD11b protein, which shows CD11b (170 kDa) and β-actin (42 kDa) immunoreactive bands, respectively. **(B)** The bar graph shows a significant increase of CD11b in the GCI+NS group with sham+NS group, and minocycline treatment remarkably reduced CD11b expression (*p < 0.05, ***p < 0.001 compared with GCI + NS, n = 10).

As the action of MIN in the brain is related to the suppression of microglia activation (Hayakawa et al., 2008; Kobayashi et al., 2013; Simon et al., 2018), we examined this efficacy in hippocampus on POD11 (**Figure 5A**). CD11b is a biomarker for microglia activation; significant differences of CD11b expression in hippocampus between the three groups were revealed by ANOVA (**Figure 5B**, *F* = 719.6786, *P*<0.001). *Post hoc* test showed robustly elevated expression of CD11b in the hippocampus in GCI mice compared to sham mice (*p* < 0.001); MIN treatment remarkably reduced hippocampal CD11b expression in GCI mice (*p* < 0.001).

**Figure 6:**
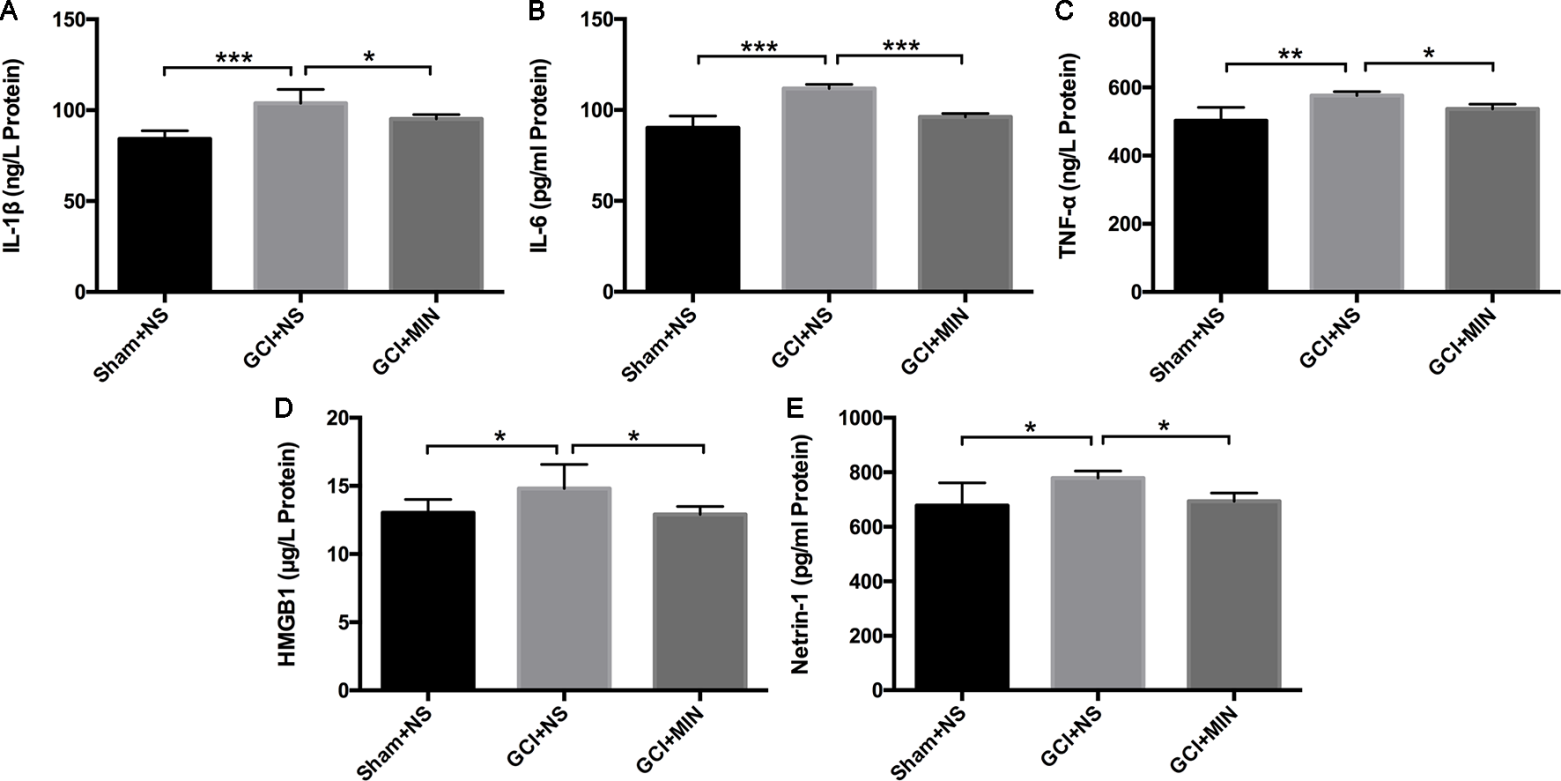
Minocycline ameliorated the levels of IL-1β, IL-6, TNF-α, HMGB1, and netrin-1 in the serum of GCI mice. Mice were treated with minocycline or saline after GCI surgery, and the serum was collected for ELISA analysis. **(A**–**E)** show the statistical results of the above inflammatory factors successively. (*p < 0.05, **p < 0.01, ***p < 0.001 compared with GCI + NS, n = 10).

IL-1β, IL-6, and TNF-α are three main pro-inflammatory cytokines released by activated microglia in the CNS. HMGB1 and netrin-1 are key factors involving in several neuroinflammatory conditions including depression. They were detected in the plasma with ELISA. One-way ANOVA indicated significant differences of IL-1β levels among the three groups (**Figure 6A**, *F* = 20.785, *P*<0.001) with the GCI + NS group the highest level. The similar statistical results were showed in the levels of IL-6, TNF-α, HMGB1, and netrin-1 among the three groups (**Figures 6B–E**, IL-6: *F* = 27.578, *P*<0.001; TNF-α: *F* = 7.802, *P* = 0.002; HMGB1: *F* = 4.008, *P* = 0.033; netrin-1: *F* = 3.938, *P* = 0.034). MIN treatment remarkably reversed GCI-induced increases of the above molecules (IL-1β: *p* = 0.012; IL-6: *p* < 0.001; TNF-α: *p* = 0.042; HMGB1: *p* = 0.018; and netrin-1: *p* = 0.043). Results indicated that MIN exerted significant anti-inflammation efficacy by reducing levels of pro-inflammatory cytokines that were released from activated microglia after GCI.

#### Minocycline Reversed the Proliferation of OPCs, But Not the Neurodegeneration Induced by GCI

**Figure 7:**
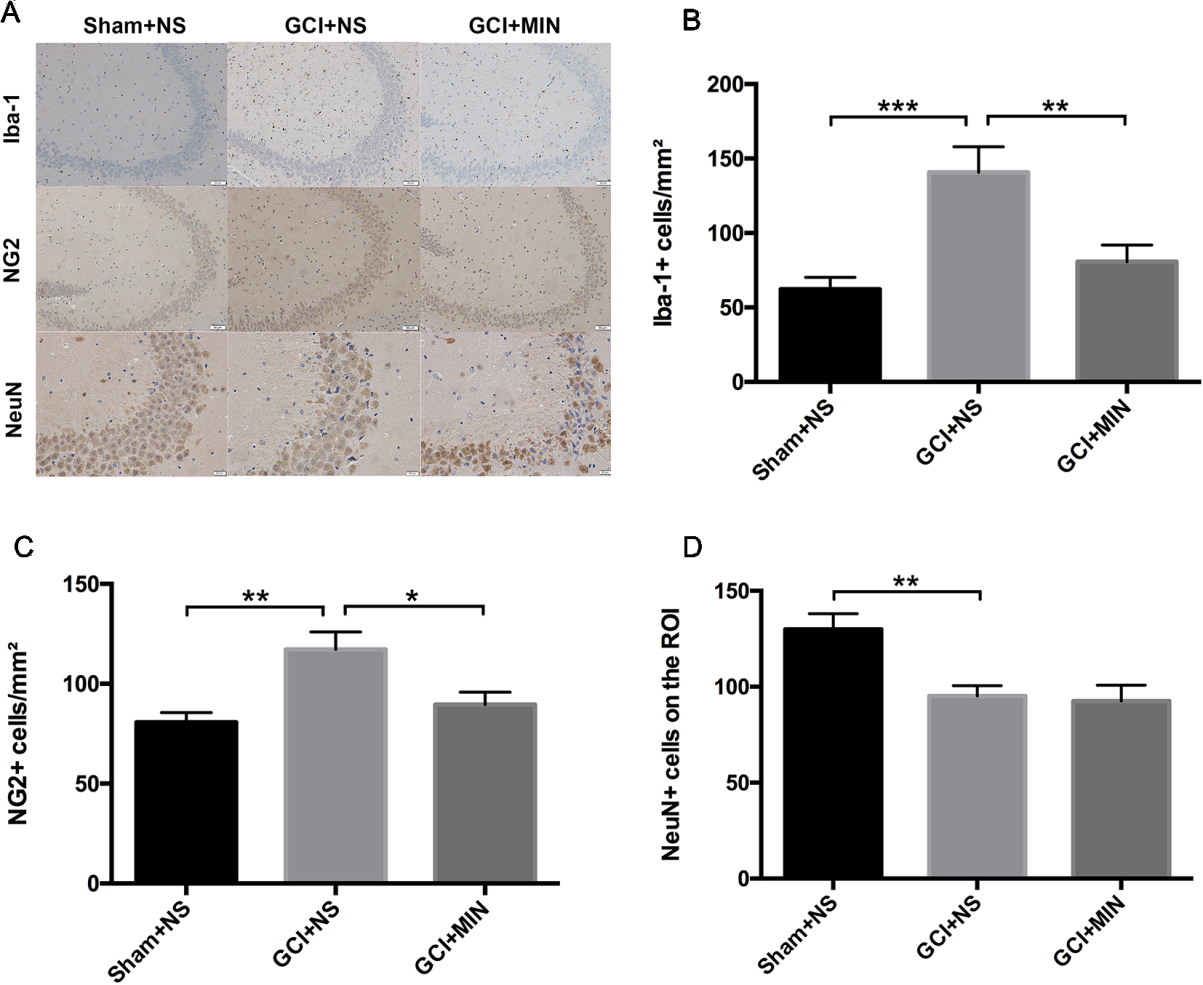
Minocycline decreased microglia and OPC activation in the hippocampus of mice conducted by GCI surgery. As revealed by immunohistochemistry of activated microglia, OPCs, and neurons in the hippocampus, minocycline administration reversed GCI-induced activation of microglia and OPCs, while no effect was observed in neurons. **(A)** shows the representative photographs of Iba-1+, NG2+, and NeuN+ staining cells in the hippocampal CA3 region. **(B**–**D)** show the above three statistical results of immunoreactive cells, respectively. (*p < 0.05, **p < 0.01, ***p < 0.001 compared with GCI + NS, n = 10).

To further investigate the effect of MIN on microglia activation and the proliferation of OLG progenitor cells (OPCs) induced by GCI, we performed immunohistochemistry staining of Iba-1 and NG2 and on POD 11 in the hippocampus (**Figure 7A**). The number of positive Iba-1and NG2 cells were counted in three visual fields (CA1, CA3, DG) of each hippocampal section for successive five sections starting from Bregma with the thickness between −1.28 mm and −2.12 mm. One-way ANOVA indicated significant differences in the number of Iba-1+ and NG2+ cells among the three groups (Iba-1: *F* = 9.951, *P* = 0.001; NG2: *F* = 7.684, *P* = 0.003). *Post hoc* comparisons revealed increased Iba-1+ (*p* < 0.001) and NG2+ (*p* = 0.001) cells in GCI mice compared to sham mice (**Figures 7B–D**), and the numbers of Iba-1+ (*p* = 0.005) and NG2+ (*p* = 0.011) cells in GCI+MIN group were significantly lower than those in GCI+NS group.

Hippocampal CA2/3 neurons are essential for spatial learning and memory (Chevaleyre and Siegelbaum, 2010). We examined the number of NeuN immunoreactive neurons in CA3 of the hippocampus. Quantification of NeuN+ cells in CA3 region revealed a significant difference between the three groups (**Figure 7D**, *F* = 8.078, *P* = 0.002). GCI+NS mice had a remarkable reduction of NeuN+ cells compared to sham+NS mice (*p* = 0.002), indicating the occurrence of neurodegeneration in hippocampus in GCI mice. However, administration of MIN did not restore the number of NeuN+ cells in CA3 region (GCI+NS *vs*. GCI+MIN: *p* = 0.797).

#### Minocycline Upregulated the Level of DA

**Figure 8:**
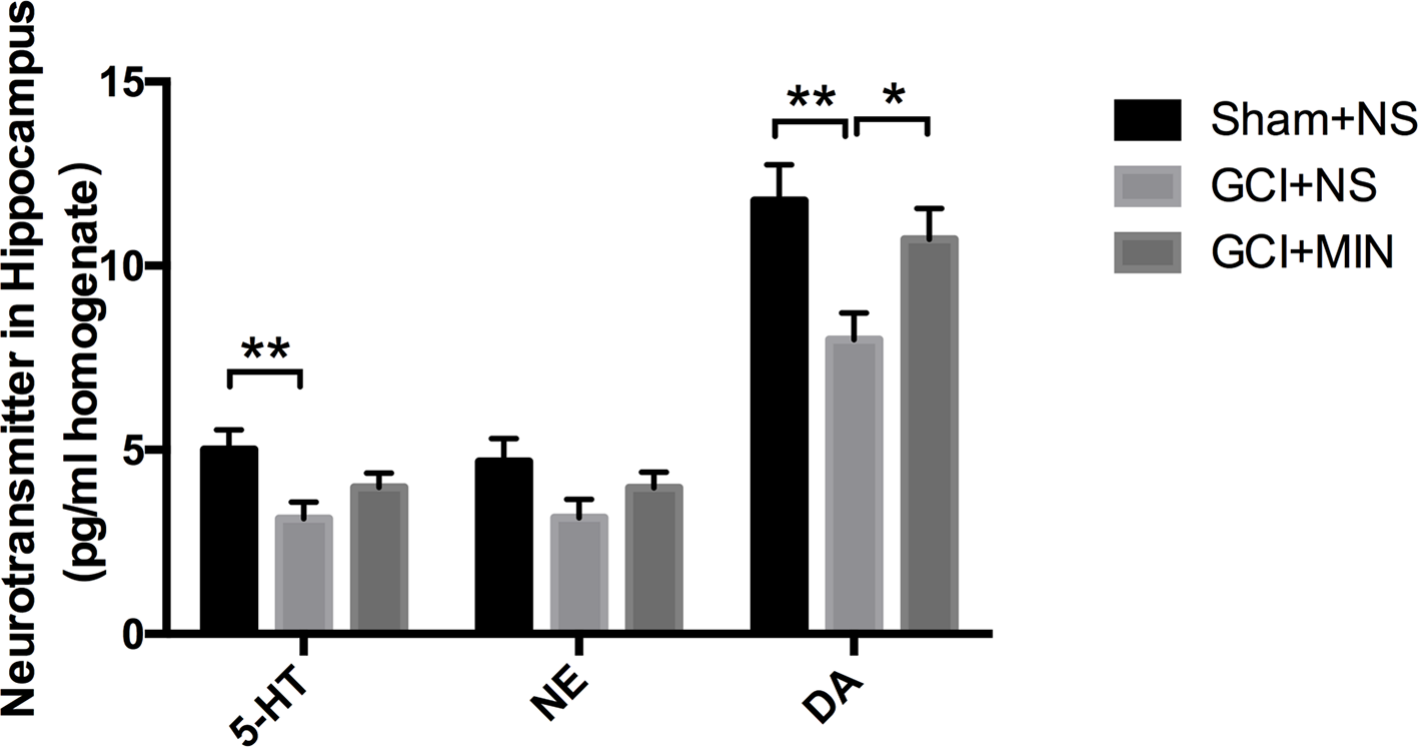
Minocycline upregulated the level of DA. Mice were sacrificed at POD11, and the hippocampus was prepared for HPLC to measure the levels of 5-HT, NE, and DA. The result indicated significant differences among the three groups of DA, while no significant influence was found of minocycline on the level of 5-HT and NE. (*p < 0.05, **p < 0.01 compared with GCI+NS, n = 10).

The contents of three monoaminergic neurotransmitters including DA, 5-HT, and NE in the hippocampus from three groups were measured by HPLC. One-way ANOVA indicated there were significant differences of DA levels among the three groups (*F* = 5,176, *P* = 0.013); *post hoc* comparisons revealed significant differences between the GCI+NS and sham+NS (*p* = 0.004) and between the GCI+MIN and GCI+NS (p = 0.033). One-way ANOVA indicated significant differences of 5-HT levels among the three groups, with the highest in sham+NS group (**Figure 8**, *F* = 4.168, *P* = 0.026). *Post hoc* comparisons revealed a significant difference between GCI+NS and sham+NS groups (*p* = 0.008) but not between the GCI+MIN and GCI+NS groups (*p* = 0.202). No differences were found in NE levels among the three groups (*F* = 2.127, *P* = 0.139). Those results showed that the level of DA in the hippocampus was upregulated by MIN treatment after the GCI. However, MIN has no significant influence on the level of 5-HT and NE in hippocampal areas.

## Discussion

Oligodendrocytes in areas of white matter are highly vulnerable to cerebral ischemia; transient GCI can induce OLGs death and white matter injury (Petito et al., 1998; Moxon-Emre and Schlichter, 2010; Uchida et al., 2010). White matter injury is a characteristic pathology in several cerebral vascular diseases including VD and VCI caused by GCI (O’ Brien et al., 2003; Mao et al., 2019). Clinical studies have found that TIA is an independent risk factor for both depression and cognitive impairment (Luijendijk et al., 2011; Broomfield et al., 2014; van Rooij et al., 2014). Ischemia-induced neuroinflammation is a leading cause of OLGs death and demyelination in the cerebral vascular diseases (Ohtaki et al., 2004; Moxon-Emre and Schlichter, 2010) and is associated with disrupted development of OPCs (Falahati et al., 2013). Microglia are innate immune cells in CNS, and microglia activation plays an essential role in white matter damage and myelination repair disorder (van Tilborg et al., 2018). Neurotransmitters play essential roles in CNS myelination. We and others showed that quetiapine, an atypical antipsychotic, enhanced OLG maturation in a GCI animal model (Belachew et al., 1999; Bi et al., 2012). Therefore, the ischemia-induced neuroinflammation may modulate myelination through affecting neurotransmitters. In this study, we examined the effect of MIN on GCI-induced depression and cognitive impairment, microglia activation, OPCs, and myelinated OLG numbers as well as neurotransmitter levels by using a BCCAO induced GCI model.

Several studies have reported similar findings to this one. Repeated transient GCI induced depressive behavior in mice, and MIN treatment significantly alleviated the depressive behavior (Yan et al., 2004; Yan et al., 2007; Molina-Hernandez et al., 2008; O’Connor et al., 2009). Additionally, MIN demonstrated antidepressant-like action in that it reduced immobility phase by increasing climb behavior, and its synergism with the antidepressant desipramine and glutamate receptor antagonists resulted in an application of subthreshold dose of both (Molina-Hernandez et al., 2008). Furthermore, in a lipopolysaccharide-induced depressive mouse model, MIN treatment significantly reduced the immobile time in TST and FST (O’Connor et al., 2009). In our study, a GCI mouse model was established by using BCCAO, which is different from other depression models in that it is more likely to be a VD model. In this model, MIN also reduced the immobile time in the two depression associated behavior tests, suggesting that MIN has an antidepressant effect on animals experiencing cerebral ischemia. However, no improvement in cognition was found after MIN administration, which was inconsistent with a previous study (Ma et al., 2015). The discrepancy may be due to the difference in surgical procedure and time of the MWM testing after surgery. What is more, the findings were interesting given the pathological changes in the hippocampus. We assumed that although there was no significant difference in cognitive function after MIN administration, there was a trend of cognitive improvement, which might be explained by pathological changes.

As to the inflammatory hypothesis of GCI, it is possible that MIN exerts an antidepressant effect through its anti-inflammation efficacy by inhibiting activation of microglia. The elevated levels of pro-inflammatory cytokines released by activated microglia have been found to be the risk of major depression in many studies (Wong et al., 2008; Baune et al., 2010; Cerri et al., 2010; Dahl et al., 2014), while antidepressant treatment successfully reduces these pro-inflammatory molecules (Miller et al., 2009). Our data have demonstrated that MIN reduced the number of activated microglia and levels of pro-inflammatory cytokines, which is consistent with other studies (Chu et al., 2007; Liu et al., 2007). Our study has observed a downregulation of netrin-1, a crucial factor for axon guidance (Croft et al., 2014), after MIN administration, supporting netrin-1 as a promising candidate for depression treatment (Zeng et al., 2017).

High-mobility group box 1 (HMGB1), also known as an alarmin protein, has been implicated as a critical factor involved in ischemia–reperfusion injury, mood disorder, cognitive impairment, and inflammation-related diseases in the CNS (Goldstein et al., 2006; Takata et al., 2012). After binding to receptors in microglia, HMGB1 activates its downstream pathways and regulates the expressions of inflammatory factors, such as IL-1β, IL-6, and TNF-α (Wang et al., 2018). A study by Hayakawa and his colleagues has reported that delayed MIN treatment ameliorated neurologic impairment *via* an HMGB1-inhibiting mechanism (Hayakawa et al., 2008). Our group has also demonstrated that exogenous administration of HMGB1 induced depression-like behavior in mice and sequential myelin injury, whereas the inhibition of HMGB1 reversed these effects (Lian et al., 2017). In addition, we found that HMGB1 level in peripheral blood of depressed patients was elevated, and this was correlated with myelin-related markers (unpublished). All the data suggested that the increased expression of HMGB1 is associated with microglia activation, myelin damage, and depressive behavior. In the present study, we have shown that MIN could significantly reverse the elevated serum HMGB1 level in GCI animals, which is in line with our hypothesis.

MIN led to substantial increases of myelin density in CC and MBP-positive mature OLGs and at same time a decrease in Iba-1+ microglia and NG2+ OPCs in the hippocampus, suggesting that reduced microglia activation and enhanced OPC maturation could be achieved by MIN treatment. Our data support a study showing that MIN could increase the number of OPCs and alleviate the apoptosis of mature OLGs in white matter in mice after chronic cerebral hypoperfusion (Ma et al., 2015). However, in cuprizone-induced demyelination model, MIN could reduce remyelination (Tanaka et al., 2013). One explanation for this is the pathophysiologic processes in the two models may be different, especially the different types of activated microglia are involved in demyelination, although demyelination in white matter occur in both models (David and Kroner, 2011). Therefore, further research needs to be done on diverse microglial functions. During the recovery phase of many CNS demyelination disorders, the differentiation of OPCs to mature OLGs plays a crucial role in rehabilitation of white matter integrity and function (Chamberlain et al., 2015; Han et al., 2015; Luo et al., 2015). Early studies revealed that dopamine plays a direct role in OPCs differentiation or the formation of myelin by mature OLGs (Bongarzone et al., 1998; Joseph and Dyer, 2003). A recent study reported that the deficiency of dopamine receptor D3 could result in depressive-like symptoms (Moraga-Amaro et al., 2014), suggesting a beneficial role of this monoamine neurotransmitter in remyelination. Our previous study found that quetiapine, an atypical antipsychotic, functions to modulate DA receptors and significantly promotes OPC maturation in the same animal model as the present study (Bi et al., 2012). In the present study, the significant increase of DA content in the hippocampus of GCI mice with MIN treatment may contribute to the amelioration of depressive behaviors. 5-HT and NE are known to be important monoamine neurotransmitters that are involved in the improvement of depressive symptoms; antidepressant agents, such as selective serotonin reuptake inhibitor (SSRI) and serotonin–norepinephrine reuptake inhibitor (SNRI), exert their therapeutic effects mainly *via* upregulating contents of 5-HT and NE in the CNS. Although depressive behavior had been ameliorated in GCI animals by MIN, the levels of 5-HT and NE in the hippocampus were not affected significantly. Our data may give a better understanding of the poor therapeutic responses of SSRI and SNRI in the treatment of vascular depression. Further research is needed to explore the underlying mechanisms by which MIN exerts its anti-depression effects through neurotransmitters.

## Data Availability Statement

The datasets generated for this study are available on request to the corresponding author.

## Ethics Statement

This study was carried out in accordance with the recommendations of the Animal Research Guidelines for the care and use of laboratory animals. The protocol was approved by the Animal Care Committee of the Second Military Medical University.

## Author Contributions

All authors have participated and made substantial contributions to this paper. BD and HL contributed to designing the study, conducting the experiments, and collecting the data. HZ and BD contributed to the data analysis and interpretation and writing of the manuscript. CF, YL, and ML performed part of data analysis. XB and YZ contributed to the study design. YZ, XB, ZW, and BD contributed to the manuscript revisions. All authors had approved the final manuscript.

## Funding

This work was supported by the National Natural Science Foundation of China (NSFC, NO.81571299).

## Conflict of Interest

The authors declare that the research was conducted in the absence of any commercial or financial relationships that could be construed as a potential conflict of interest.
